# Help-Seeking on Facebook Versus More Traditional Sources of Help: Cross-Sectional Survey of Military Veterans

**DOI:** 10.2196/jmir.9007

**Published:** 2018-02-26

**Authors:** Alan R Teo, Heather E Marsh, Samuel B L Liebow, Jason I Chen, Christopher W Forsberg, Christina Nicolaidis, Somnath Saha, Steven K Dobscha

**Affiliations:** ^1^ Health Services Research and Development Center to Improve Veteran Involvement in Care Department of Veterans Affairs Portland Health Care System Portland, OR United States; ^2^ Department of Psychiatry Oregon Health & Science University Portland, OR United States; ^3^ School of Public Health Oregon Health & Science University and Portland State University Portland, OR United States; ^4^ Department of Medicine Oregon Health & Science University Portland, OR United States; ^5^ Regional Research Institute School of Social Work Portland State University Portland, OR United States; ^6^ Department of Medical Informatics and Clinical Epidemiology Oregon Health & Science University Portland, OR United States

**Keywords:** social media, social networking sites, internet, Facebook, service use, utilization, treatment-seeking

## Abstract

**Background:**

The media has devoted significant attention to anecdotes of individuals who post messages on Facebook prior to suicide. However, it is unclear to what extent social media is perceived as a source of help or how it compares to other sources of potential support for mental health problems.

**Objective:**

This study aimed to evaluate the degree to which military veterans with depression use social media for help-seeking in comparison to other more traditional sources of help.

**Methods:**

Cross-sectional self-report survey of 270 adult military veterans with probable major depression. Help-seeking intentions were measured with a modified General Help-Seeking Questionnaire. Facebook users and nonusers were compared via *t* tests, Chi-square, and mixed effects regression models. Associations between types of help-seeking were examined using mixed effects models.

**Results:**

The majority of participants were users of social media, primarily Facebook (n=162). Mean overall help-seeking intentions were similar between Facebook users and nonusers, even after adjustment for potential confounders. Facebook users were very unlikely to turn to Facebook as a venue for support when experiencing either emotional problems or suicidal thoughts. Compared to help-seeking intentions for Facebook, help-seeking intentions for formal (eg, psychologists), informal (eg, friends), or phone helpline sources of support were significantly higher. Results did not substantially change when examining users of other social media, women, or younger adults.

**Conclusions:**

In its current form, the social media platform Facebook is not seen as a venue to seek help for emotional problems or suicidality among veterans with major depression in the United States.

## Introduction

Social media has become an integral part of people’s daily lives, including military veterans. As of 2016, there were 1.89 billion active users on Facebook [1], with 76% logging on daily and spending 50 minutes on average on the site [2,3]. All told, an estimated 1 out of every 7 minutes spent online is spent on Facebook [4]. In 2014, Facebook reported that 4 million of its users were US active duty members or military veterans [5], and a 2012 online survey on personal technology use among military service members found that more than three-quarters of them used social networking sites, predominantly Facebook [6].

Facebook is most commonly used for purposes such as entertainment, communicating with friends and family, and keeping up with news and current events, with 31% to 47% of users citing such reasons [7]. However, there are also ways Facebook appears to be used that may be relevant to obtaining emotional support or help for mental health concerns. For instance, 30% of Facebook users cite learning about ways to help others as a reason for their Facebook use, and 23% note receiving support from network members as another reason [7]. Among veterans, US Department of Veteran Affairs (VA) patients appear to use social media to find others with similar health problems at about the same rate as nonveterans [8].

Social media may be a valuable venue for help-seeking for several reasons. It is a highly accessible resource, and use of internet-based sources of help for health issues is common [9]. Researchers have been interested in the potential to use social media to reach, recruit, identify, engage, support, or treat individuals at risk for mental health problems [10-15]. Individuals coping with mental illness are active on social media [16], and Facebook is used as a place to share feelings [17] and mobilize social support [18], particularly advice and practical help [19]. Occasionally, it is even used as a forum to disclose suicidal thoughts [20]. Facebook, for its part, has collaborated with suicide prevention organizations to develop tools to identify and intervene on behalf of individuals who appear to be at risk for self-harm or suicide [21].

The importance of novel approaches to enhance help-seeking is accentuated by the fact that rates of help-seeking are generally low in proportion to the number of people suffering from psychiatric problems such as depression [22] and even recent suicidal ideation [23]. A similar pattern is seen among military service members and veterans, who are also thought to underuse mental health services or be underserved [24-26]. The need to address this gap between suffering and help-seeking is likely to grow given evidence of rising suicide rates and the list-topping proportion of the global burden of disease attributable to mental illnesses [27,28]. However, it is unclear to what extent social media actually serves as an existing source of help-seeking, particularly for mental health problems and particularly beyond the adolescent and young adult population [29,30].

Thus, the aim of this study was to evaluate the degree to which veterans with depression use social media for help-seeking in comparison to other more traditional sources of help.

## Methods

### Recruitment

We drew participants from a larger study of 301 primary care patients at a VA hospital and its satellite clinics who had symptoms of major depression and reported having at least one close relationship. Patients were excluded if they had severe hearing impairment or recently active major psychiatric comorbidities (bipolar disorder, psychosis, or neurocognitive disorder). Due to the low percentage of women veterans, we oversampled women to increase diversity of our sample. Potentially eligible veterans were first screened for depression via a phone-administered 8-item Patient Health Questionnaire (PHQ-8) followed by an in-person visit if eligible and interested. Those eligible for inclusion in this study were the 270 individuals who had a PHQ-8 score ≥9, who were then administered a set of survey items about social media use. Meta-analyses have found that a cutoff score of 9 on the PHQ-9 is one of the optimal choices for diagnosing major depression in primary care settings [31]; the identical cutpoint should be used on the PHQ-8 due to its high level of correlation with PHQ-9 score [32].

### Measures

#### Help-Seeking

We administered the General Help-Seeking Questionnaire (GHSQ), an adaptable self-report measure that assesses intention to seek help if experiencing an emotional problem or suicidal thoughts. Multiple possible sources of help are presented, and response options for each range from 1=extremely unlikely to 7=extremely likely. Because original items were developed in Australia, we slightly modified them for an American context and added Facebook as another potential source of help. Participants were asked about help-seeking on Facebook generally, without specifying particular areas or functions within Facebook (eg, support groups or interest pages). We sorted sources into 4 categories: (1) Facebook help-seeking; (2) informal help-seeking, which is drawn from members of one’s social network such as friends and family; (3) phone helpline help-seeking, which cited the Veteran’s Crisis Line as an example; and (4) formal help-seeking, which included a mental health professional or primary care provider. For categories with multiple items, we averaged participant responses to each source. The GHSQ has strong reliability and validity [33], particularly when used in multi-item form as we did [34].

#### Social Media Use

We used a series of survey items adapted from questions used by the Pew Research Center [35,36] to assess social media use. First, we assessed whether veterans were social media users (“Do you ever use the internet or a mobile app to use Twitter, Instagram, Pinterest, Tumblr, or Facebook?”). Next, we assessed frequency of use of the same social media platforms. Finally, we assessed frequency of active social media use (sharing, posting, or commenting) because prior research has indicated that social media use is often characterized by passive consumption of information (eg, scrolling through news feeds), a behavior linked to worsened emotional well-being [37].

#### Covariates

We assessed additional sociodemographic and clinical characteristics including age, gender, minority status, education level, rurality of residence, stability of housing, financial hardship, history of suicide attempt, alcohol misuse, posttraumatic stress disorder symptoms, depression symptoms, and offline social contact. See Multimedia Appendix 1 for additional details.

### Statistical Analysis

We first examined missingness of data; all survey items had less than 2% missing data. We used mean imputation for missing responses to GHSQ items that were part of informal or formal help-seeking. We summarized key variables using descriptive statistics. We analyzed sociodemographic and clinical characteristics of participants who were Facebook users and nonusers using 2-sample *t* tests and Pearson Chi-square tests. To compare help-seeking intentions from different GHSQ source categories, we used multilevel mixed-effects linear regressions. We performed subgroup analyses to determine whether the pattern of help-seeking intentions differed when the sample was restricted to various groups of participants, including frequent Facebook users (defined as visiting at least daily), active Facebook users (defined as sharing, posting, or commenting on Facebook at least daily), and users of social media platforms other than Facebook. Analyses were performed using Stata versions 14.2 and 15.0 (StataCorp LLC), and 2-sided statistical significance was defined as *P*<.05.

## Results

### Descriptive Data

In terms of help-seeking intentions, 58.1% (157/270) endorsed having at least 1 source they intended to use (GHSQ score of 5 or more) if experiencing an emotional problem. If having suicidal thoughts, 55.6% (150/270) endorsed having at least 1 help-seeking source.

Of our 270 participants, 162 (60.0%) were Facebook users, 106 (39.3%) used Facebook at least daily, and 65 (24.1%) used Facebook actively (as opposed to passively) at least daily. Use of any social media platform other than Facebook was uncommon (49/270, 18.1%), with use of individual platforms (eg, Twitter) even more rare. Compared to nonusers, Facebook users were younger and used other social media and more likely to be women, live in urban areas, and be at risk for misusing alcohol; they were less likely to have a history of a suicide attempt (Table 1). However, mean help-seeking intentions for each source of help did not differ significantly between Facebook users and nonusers. This was true for both help-seeking for emotional problems and suicidal thoughts. Results also remained consistent after adjustment for potential confounders (age, gender, urbanicity, alcohol use, use of social media sites other than Facebook, and history of suicide attempt). All remaining results are based on data from only participants who were Facebook users.

**Figure 1:**
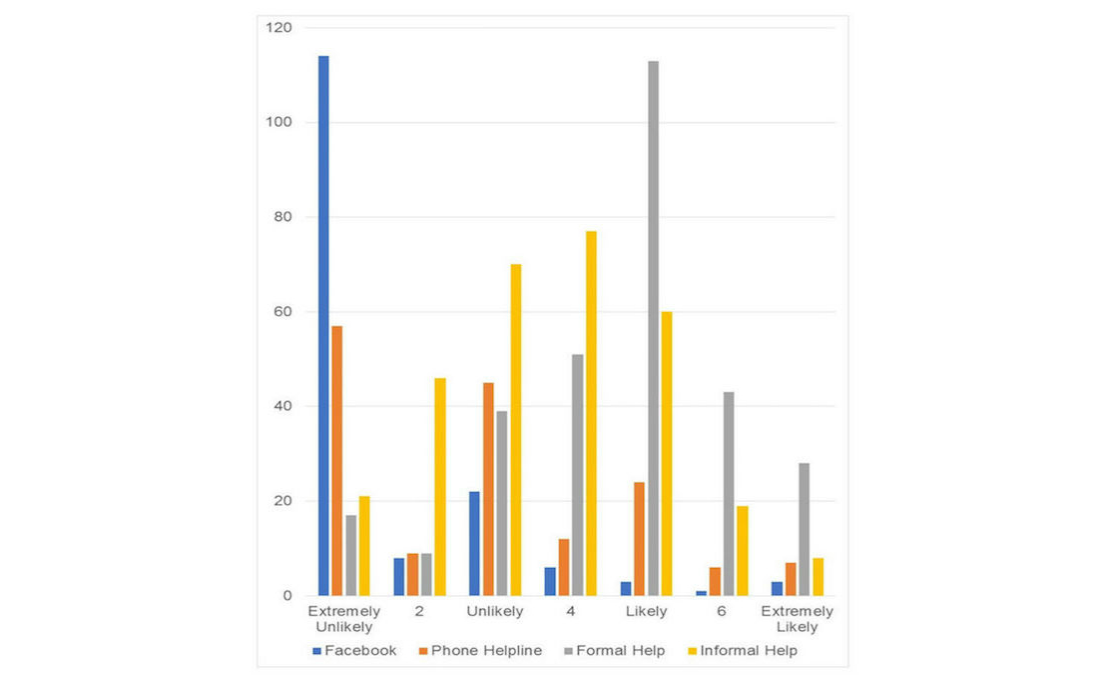
Frequency of help-seeking intentions if experiencing an emotional problem across 4 potential sources of support among Facebook users (n=162).

**Figure 2:**
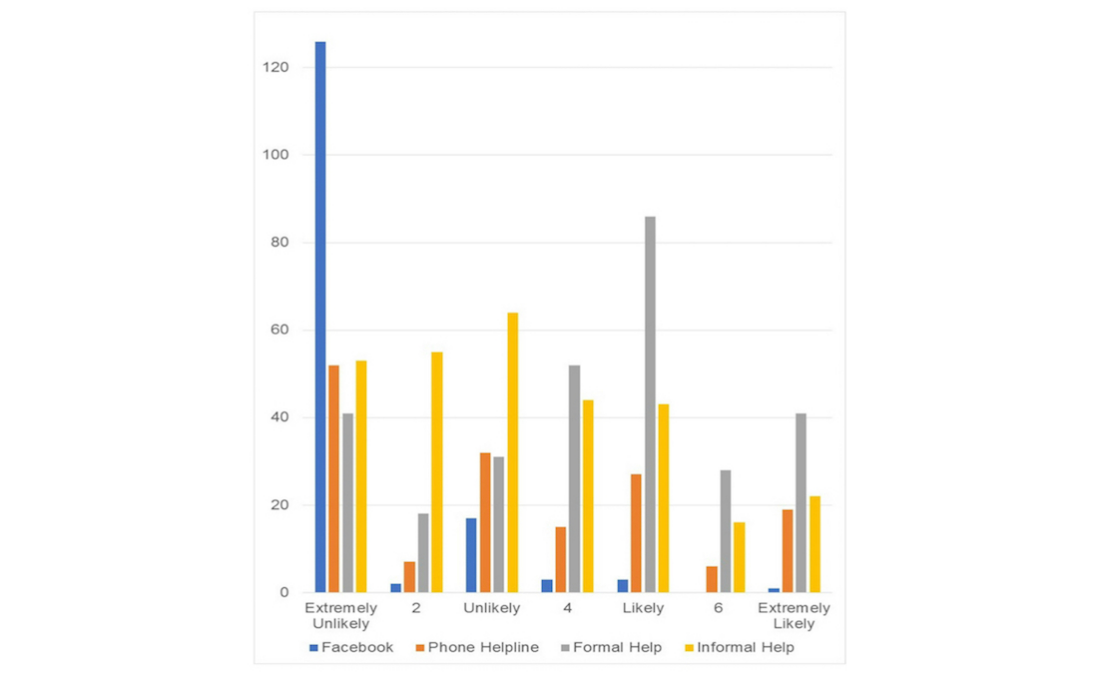
Frequency of help-seeking intentions if experiencing suicidal thoughts across 4 potential sources of support among Facebook users (n=162).

A total of 58.6% (95/162) of Facebook users endorsed having at least 1 help-seeking source for emotional problems; for suicidal thoughts, 50.6% (82/162) intended to use at least 1 source of help. The mean help-seeking intention for emotional problems via Facebook was 1.67 (95% CI 1.46 to 1.87) on a scale ranging from 1=extremely unlikely to 7=extremely likely. Help-seeking intention for suicidal thoughts via Facebook was similarly low (mean 1.41, 95% CI 1.25 to 1.58). Figures 1 and 2 illustrate the preponderance of participants who were extremely unlikely to seek help from Facebook as opposed to a more even distribution of likelihood to seek help from other sources.

### Comparisons of Help-Seeking Intentions by Source of Help

**Figure 3:**
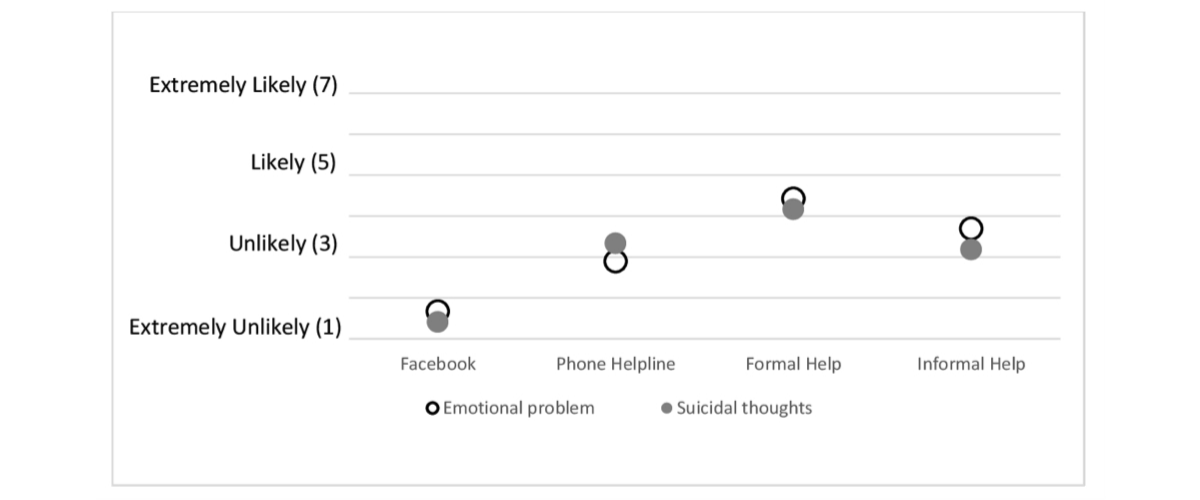
Help-seeking intentions for 4 potential sources of support among participants who use Facebook (n=162).

As shown in Figure 3, comparisons of help-seeking intentions through Facebook versus other potential means of help (formal, informal, or phone helpline) revealed highly significant differences, with veterans being less likely to seek help from Facebook than from any other source. This was true for help-seeking for both emotional problems and suicidal thoughts. For example, the mean level of help-seeking intentions via a phone helpline was significantly higher for both emotional problems (2.89; 95% CI 2.61 to 3.17, *P*<.001) and suicidal thoughts (3.33; 95% CI 3.00 to 3.66, *P*<.001) compared to Facebook. In addition, the mean level of help-seeking intentions was highest for formal sources for both emotional problems (4.42; 95% CI 4.20 to 4.65, *P*<.001) and suicidal thoughts (4.16; 95% CI 3.88 to 4.44, *P*<.001). Detailed data on comparisons of different help-seeking sources is contained in Multimedia Appendix 1.

### Help-Seeking Intentions Among Subgroups of Facebook Users

Results were mostly similar when we restricted analyses to participants who were frequent Facebook users (106/162) and active Facebook users (65/162) (see figures in Multimedia Appendix 1). Active Facebook users did show a small but significant 0.53-point increase (95% CI 0.12 to 0.94, *P*=.01) in intention to seek help from Facebook for emotional problems and a 0.33-point increase (95% CI 0.01 to 0.66, *P*=.047) in intention to seek help from Facebook for suicidal thoughts, compared to nonactive Facebook users. Among Facebook users who also used other forms of social media (42/162), help-seeking intentions via Facebook were not significantly different from those who exclusively used Facebook, either for emotional problems (1.90 vs 1.59; 95% CI –0.15 to 0.78, *P*=.18) or suicidal thoughts (1.58 vs 1.36; 95% CI –0.15 to 0.59, *P*=.25).

In examination of demographic groups of interest, results were again consistent with the overall sample. Among women (28/162), help-seeking intentions via Facebook were not significantly different from men for emotional problems. Finally, among participants younger than age 40 years (37/162), help-seeking intentions via Facebook were not significantly different from participants age 40 years and over, either for emotional problems or suicidal thoughts.

**Table 1:** Characteristics of participants with active depressive symptoms.

Characteristic			Facebook users (n=162)		Facebook nonusers (n=108)		*P* value	
**Age, years, mean (SD)**			52 (14)		61 (13)		<.001	
		Under 40, n (%)		37 (22.8)		11 (10.2)		
		40-49, n (%)		29 (17.9)		8 (7.4)		
		50-59, n (%)		40 (24.7)		20 (18.5)		
		60 or over, n (%)		56 (34.6)		69 (63.9)		
Male, n (%)			134 (82.7)		99 (91.7)		.04	
Racial or ethnic minority, n (%)			37 (22.8)		26 (24.1)		.81	
**Education, n (%)**							.55	
	High school or less		20 (12.3)		20 (18.5)			
	Some college		59 (36.4)		39 (36.1)			
	Two-year degree		43 (26.5)		25 (23.1)			
	Four-year degree or more		40 (24.7)		24 (22.2)			
Rural residence, n (%)			22 (13.6)		25 (23.1)		.04	
Stable housing, n (%)			149 (91.2)		98 (90.7)		.72	
Financial hardship, n (%)			44 (27.2)		28 (25.9)		.97	
History of suicide attempt, n (%)			19 (11.7)		29 (26.9)		.001	
Alcohol misuse^a^, n (%)			65 (40.1)		27 (25.0)		.01	
PTSD symptoms^b^, n (%)			108 (66.6)		67 (62.0)		.44	
PHQ-9 score^c^, n (%)			15 (9.3)		15 (13.9)		.77	
Use social media besides Facebook^d^, n (%)			42 (25.9)		7 (6..5)		<.001	
**Offline social contact^e^, n (%)**							.57	
	Every few months or less		15 (9.3)		7 (6.5)			
	Once or twice a month		35 (21.6)		18 (16.7)			
	Weekly to a few times a week		79 (48.8)		57 (52.8)			
	Daily or more		33 (20.4)		26 (24.1)			

^a^Alcohol misuse was operationalized as an Alcohol Use Disorders Identification Test score ≥4 in men (≥3 in women).

^b^Posttraumatic stress disorder was operationalized as a Primary Care Posttraumatic Stress Disorder Checklist score ≥3.

^c^Nine-item Patient Health Questionnaire score represents severity of symptoms of major depression.

^d^Participants were asked whether they ever use Instagram, Pinterest, Tumblr, or Twitter.

^e^Average frequency of in-person social contact with up to 3 individuals nominated as close relations.

We identified a small subgroup (9/162) of participants who indicated intention to seek help for emotional problems or suicidal thoughts from Facebook (GHSQ score of 5 or more). These individuals had somewhat higher levels of help-seeking intentions more generally. Their mean help-seeking intentions using formal, informal, and phone sources for either emotional problems or suicidal thoughts was 4.83, which was 1.29 points (95% CI 0.45 to 2.13, *P*=.003) higher than other Facebook users.

## Discussion

### Principal Findings

The primary finding from this study is that social media appears to be a very undesirable venue for mental health help-seeking for VA patients with probable major depression. This finding remained true even among the most frequent users of Facebook and among individuals who used Facebook in an active way (which is more associated with positive mental health than passive use) [38]. Findings were also consistent whether the reason for help-seeking was a more mild emotional concern or something much more severe (suicidal thoughts).

The lack of interest in Facebook for help-seeking is all the more striking when compared to higher levels of interest in offline sources of help. The majority of our military veterans endorsed at least one venue they were likely to use for help-seeking. Mean help-seeking intentions for formal help and phone helplines in our sample were higher than in a prior study of Iraq/Afghanistan-era military veterans [39] and similar to prior studies involving Australian college students [40].

This study does not reveal why individuals do not intend to seek help from Facebook. However, we suspect several possible reasons for the large discrepancy in the likelihood of informal help-seeking via Facebook versus offline sources. First, many “Facebook friends” are not “real friends,” or at least not close ones. Prior empirical work has shown that only a small segment of Facebook friends are actually close, personal social connections [41]. It is this small group that is most likely to influence behavior in the real world [41]. Second, it may be stigmatizing to self-disclose mental health problems on Facebook. People have a strong tendency to present a positive self-image on Facebook [42], and stigma is a major barrier to help-seeking that occurs disproportionately among military veterans [43]. Third, interactions on Facebook are likely perceived as being more impersonal and lacking a human touch. So-called “broadcast communication,” which includes posts such as status updates that are sent out widely to Facebook friends [44], predominates on Facebook and perhaps contributes to such a perception. Fourth, participants may not have perceived Facebook as a trustworthy platform in which to openly discuss mental health matters. Concerns about privacy might stifle mental health help-seeking, and some research has suggested trust in social media is low in the United States and acts as a predictor of whether one engages in self-disclosure of health issues online [45]. There are areas within Facebook that are less susceptible to concerns related to impersonal communication and anonymity. For example, closed support groups, including ones tailored to veterans, would likely be more socially acceptable forums within Facebook for help-seeking or exchange of social support [46]. Last, because many of the most common reasons for using Facebook are unrelated to health concerns, it may be that, for military veterans, Facebook simply does not come to mind as a help-seeking tool.

In addition, help-seeking intentions via Facebook may be more favorable in individuals younger than our sample population, such as millennials who have grown up with Web 2.0 and social media [47]. Prior research has even shown that adolescents may disclose more personal information on social media than in person [48]. It is also encouraging that when individuals do self-disclose negative emotions on Facebook, they often receive objective social support, even more so if the person is depressed [49]. Researchers are also developing tools to help detect risk for suicide based on content of social media posts, which could in theory lead to ways to reach out to individuals online even if they do not actively seek out help themselves [50].

### Limitations

The primary limitation of this study is the lack of measurement of actual help-seeking or treatment utilization, given that research has found stated intentions of help-seeking may not translate into actual help-seeking behaviors [51]. Likewise, we were unable to capture whether veterans ever posted on Facebook in the act of help-seeking. Although emotional problems and even suicidality were relatively common in our sample, results must be interpreted cautiously as our survey queried intentions to seek help in a hypothetical scenario. Additionally, survey order effects could have influenced results such that individuals underreported Facebook help-seeking intentions. Because the item on Facebook help-seeking intentions was placed after items on other sources of help such as friends and family, respondents may have perceived Facebook as being mutually exclusive from these options. Finally, results are not likely generalizable to other populations not well represented in our sample of military veterans, including veterans who lack any social supports, women, or youth.

### Conclusions

Our results should not be interpreted as negating the relevance of tools, such as one recently rolled out by Facebook [28], to assist individuals actively experiencing suicidal ideation. Rather, we believe our results frame the utility of such tools as being best suited to the select few who both experience suicidality and are comfortable using Facebook in a crisis. Overall, this study suggests Facebook in its current form is by no means perceived as a go-to source for mental health help-seeking among veterans with depression. Instead, more traditional sources of support appear to be the most viable venues for help-seeking in this population.

## Appendix

Multimedia Appendix 1 Sociodemographic and clinical variables examined.

## Abbreviations

GHSQ: General Help-Seeking Questionnaire
PHQ-8: Patient Health Questionnaire
PTSD: Posttraumatic stress disorder
VA: US Department of Veterans Affairs
