# Supplementary material for: Help-Seeking on Facebook Versus More Traditional Sources of Help: Cross-Sectional Survey of Military Veterans
Source: J Med Internet Res. 2018 Feb 26;20(2):e62. doi: 10.2196/jmir.9007 (PMC5847817; doi:10.2196/jmir.9007)
Supplement: Multimedia Appendix 1 [file jmir_v20i2e62_app1.pdf]

Online Supplement

The following is a list of sociodemographic and clinical variables that were examined. Variables are drawn from responses to survey items.

Age: Measured in years.

Gender: Male, female, or “a gender not listed here.”

Minority status: This was assessed with a score of ‘0’ for ‘White, non-Hispanic’ and a score of ‘1’ if respondents indicated some other race (‘African American,’ ‘Native American,’ ‘Asian, or ‘Other’), ‘Multiple races’ or ‘White, Hispanic.’

Education level: This was assessed by participants responding ‘some high school,’ ‘GED or high school diploma,’ ‘some college,’ ‘two-year college degree (associate’s degree),’ and ‘four-year college degree or higher’ to “What is your highest level of education?”

Rurality of residence: This is based on administrative data of participants residing in a location geocoded as ‘rural.’

Stability of housing: This was assessed with a self-report measure on whether respondents had, in the past 2 months, been living in stable housing (owning, renting, or staying in as part of a household).

Financial hardship: This was assessed by participants responding ‘somewhat difficult,’ ‘difficult,’ or ‘very difficult’ to paying for the very basics like food, housing, medical care, and heating.

History of suicide attempt: This was assessed by answering ‘yes’ to ‘In your lifetime, have you ever had a suicide attempt?’

Alcohol misuse: This was assessed with the Alcohol Use Disorders Identification Test-Consumption Questions (AUDIT-C), which is a 3-item self-report scale that identifies hazardous drinkers or who have active alcohol use disorders (alcohol abuse and dependence). Scores range from 0-12, and a score of 4 or greater (3 in women) is considered positive for identifying hazardous drinking.

Post-traumatic stress disorder symptoms: This was assessed with the self-report Primary Care Post-Traumatic Stress Disorder Screen (PC-PTSD), a 4-item self-report scale designed for use in primary care and other medical settings. Scores range from 0-4, and a score of 3 or greater is a ‘positive’ PTSD screen.

Depression symptoms: Depression symptoms were assessed at two timepoints: phone screening via the eight-item Patient Health Questionnaire (PHQ-8) and at an in-person study visit with the nine-item Patient Health Questionnaire (PHQ-9). Results here are from the in-person study visit. The latter has a score range of 0 to 27 and assesses presence of major depressive disorder symptoms over the past two weeks.

Offline social contact: This was assessed with a self-report measure of how much in-person social contact one has with their confidants: ‘on average, how often do you do each of the

following with [Person 1]: talk in person?' Individual items are scored from 1 to 6, with statements ranging from 'Once or twice a year or never,' to 'Once a day or more.' The higher the score, the more frequent in-person contact one has.
